# Lesions involving the outer surface of the bone in children: a pictorial review

**DOI:** 10.1007/s13244-016-0527-0

**Published:** 2016-10-19

**Authors:** Apeksha Chaturvedi, R. A. C. Dilhani Ranasinghe, Abhishek Chaturvedi, Steven P. Meyers

**Affiliations:** 1 Department of Imaging Sciences, University of Rochester Medical Center, 601 Elmwood Ave, P.O. Box no. 648, Rochester, NY 14642 USA; 2 Department of Radiology, Teaching Hospital, Kandy, Sri Lanka

**Keywords:** Cortical/juxtracortical osseous lesions, Periosteal abnormalities, Radiograph, MRI

## Abstract

**Background:**

Lesions involving the outer cortical surface of the bone occur quite often among children. Broadly, these include benign cortical, juxtacortical and periarticular lesions, dysplasias affecting the cortical bone, regional and diffuse periosteal pathology and malignant tumours. Some of these lesions are unique to the paediatric population; others are more frequently seen among children than adults — yet others have an adult predilection but can occasionally be seen in children.

**Methods:**

A complete list of differential considerations for lesions involving the outer cortical surface of the bone in children is presented. Imaging characteristics on plain film radiography and MR are described in association with multiple examples and illustrations.

**Conclusion:**

A pictorial review detailing the imaging features of surface lesions of the bone in children will be a useful aide for both radiologists and their clinical colleagues, and will help them sort their way through the maze of differential diagnoses for these abnormalities.

*Teaching Points*

*• Surface lesions of bones in children comprise a distinct entity and differ from those in adults*.

*• Imaging plays an important role towards classifying surface lesions of bones in children*.

*• MRI features may be characteristic and aid precise diagnosis, thus guiding further management*.

## Introduction

Surface lesions of bone have many aetiologies and manifestations. To start with, it is necessary to define this category of lesions. Broadly, any lesion, which either abuts or arises from the outer cortical or periosteal surface of the bone can be included.

The overwhelming majority of described surface lesions of the bone across age groups have at least occasionally been reported in children. This adds an element of confusion to the task of doing a mostly paediatric review. In the interest of conciseness, the current review will focus mostly on lesions, which occur more commonly, or at least comparably in children relative to adults.
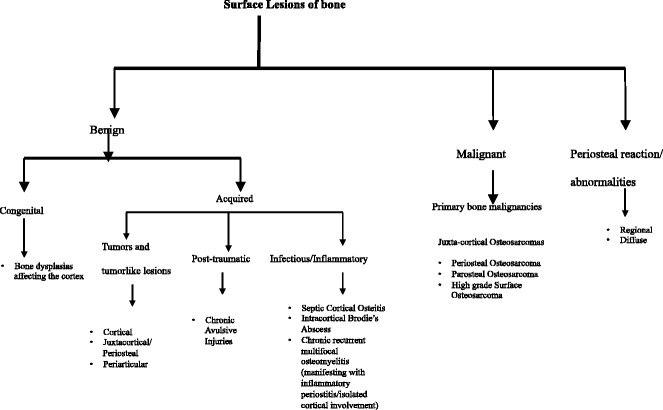


**Fig. 1:**
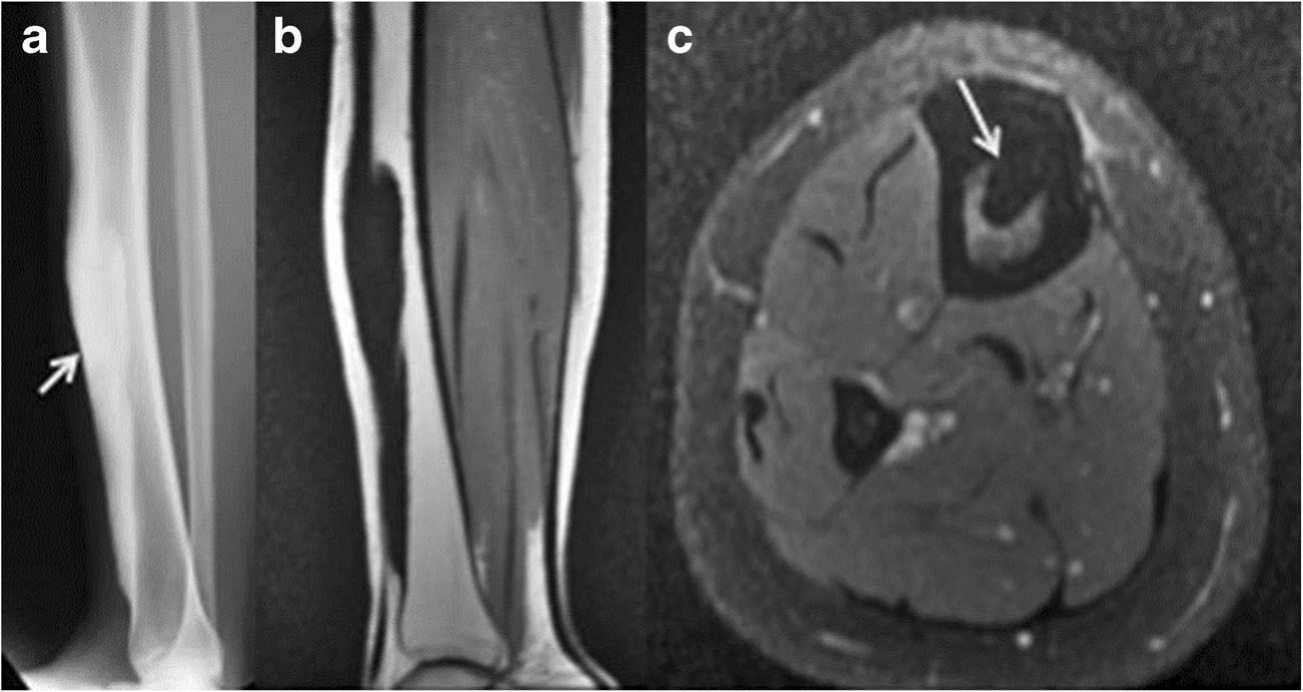
Melorheostosis along the antero-medial surface of right tibia. Antero-posterior radiograph of right tibia **a** shows “dripping candle wax” appearance (*thick arrow*) along the anterior cortex. **b** Coronal T1 and **c** axial fat-suppressed T2 images reveal homogenous hypointensity through the lesion, implying mineralized osteoid matrix (*thin arrow* on **c**)
        MRI signal varies based on the relative proportions of mineralized osteoid, chondroid, and soft-tissue components in these lesions. Mineralized osteoid zones along bone cortex typically have a low signal on T1WI and T2WI, no Gd-contrast enhancement. Soft-tissue lesions may also occur adjacent to the cortical lesions, which have a mixed signal on T1WI and T2WI [2].
      Camurati–Engelmann disease (CED):
        This is a rare autosomal-dominant type of progressive diaphyseal dysplasia belonging to the group of craniotubular hyperostosis. The disorder is characterized radiologically by symmetric, bilateral cortical thickening involving the diaphyses of long bones — starting at the femurs and tibiae but ultimately involving fibulae, humeri, and forearm bones. Metaphyses may be affected during the course of the disease but epiphyses are typically spared. The skull base may be sclerotic. Disease onset is usually during childhood [3].
      Ribbing disease:
        This is another rare sclerosing bone dysplasia, which manifests in young adults; the symptoms first present after puberty (Fig. 2). Radiologically, there is asymmetric, benign endosteal and periosteal bone growth confined to long bone diaphyses and limited to lower extremities [4].

Conceptually, these surface lesions can be categorized as under:

1. Benign:
  - Congenital
    Bone dysplasias affecting the cortex
      Melorheostosis
      Camurati–Engelmann disease
      Ribbing disease
      Kenny–Caffey syndrome
  - Acquired
    - Tumours and tumour-like lesions
      - Cortical
        Osteochondroma
        Subperiosteal osteoid osteoma
        Fibrous cortical defect
      - Juxtacortical
        Juxtacortical/Periosteal chondroma
        Benign Parosteal osteochondromatous proliferation
        Dysplasia epiphysealis hemimelica
        Heterotopic ossification
      - Periarticular
        Ganglion cysts
    - Post-traumatic
      Chronic avulsive injuries
    - Infectious/Inflammatory
      Septic cortical osteitis
      Intracortical Brodie’s abscess
      Chronic recurrent multifocal osteomyelitis (manifesting with inflammatory periostitis or isolated cortical involvement)
2. Malignant:
  - Primary “surface” bone malignancies
    Periosteal osteosarcoma
    Parosteal osteosarcoma
    High-grade surface osteosarcoma
3. Periosteal reaction/abnormalities:
  - Regional
    Non-interrupted
    Multilamellated
    Solid
    Spiculated
  - Diffuse
    Infantile cortical hyperostosis (Caffey’s disease), hypervitaminosis A, hypovitaminosis C, hemophiliac pseudotumor

1. Benign:
  - Congenital
    Bone dysplasias affecting the cortex
      Melorheostosis:
        Melorheostosis is a rare bone dysplasia usually discovered in childhood; this shows a 1:1 male–female ratio [1]. This manifests as cortical hyperostosis in one or multiple bones, with intervening soft-tissue calcification or ossification [1].
        Cortical thickening of melorheostosis has a “flowing candle wax” configuration (Fig. 1). Lesions have a periosteal/endosteal epicenter and more commonly affect the lower extremity than the upper. Associated soft-tissue masses occur in approximately 25 %. The soft-tissue lesions often contain mixtures of chondroid material, mineralized osteoid, and fibrovascular tissue.

**Fig. 2:**
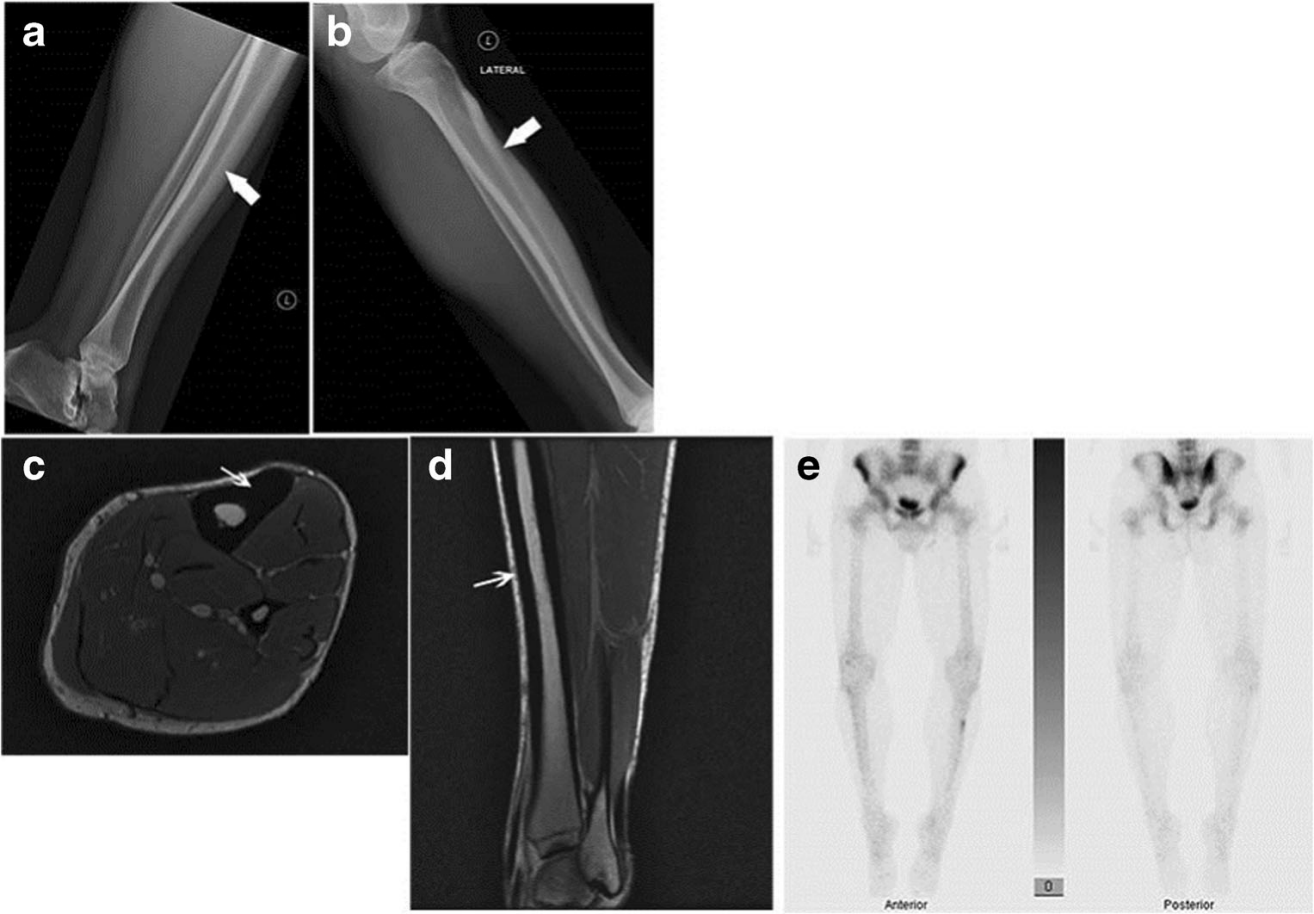
Ribbing disease. Lateral radiograph of right tibia **a** shows smooth thickening along the anterior cortex; this radiograph was obtained when the patient was 19 years of age. 7 years later, a follow-up lateral radiograph obtained as a young adult **b** shows further increased thickening along the anterior tibial cortex with narrowing of the medullary cavity. Axial **c** and sagittal **d** PD MR images of the tibia obtained at the same time as the second radiograph reveal smooth cortical thickening anteriorly. No unusual abnormal uptake of radiotracer was noted on the concomitantly performed Tc-99m MDP labeled bone scan **e**
      Kenny–Caffey syndrome (KCS):
        Kenny–Caffey syndrome (KCS) is a very rare dysmorphologic syndrome. The hallmark of this syndrome is a proportionately short stature, cortical thickening and medullary cavity obliteration/narrowing of tubular bones, delayed closure of anterior fontanelle, eye abnormalities, and hypoparathyroidism [5].
  -Acquired
    - Tumours and tumour-like lesions
  - Cortical
    Osteochondroma
    Osteochondromas constitute the commonest bone tumours in children. Overall, these account for 10 to 15 % of overall bone tumours and 20–50 % of benign bone tumours [6, 7]. Up to 75 % of patients are younger than 20 years.
    These can be solitary or multiple; the latter condition is referred to as diaphyseal aclasis or hereditary multiple exostosis (HME). Proposed aetiologies range from developmental origin to true neoplasms [8]. The majority of these lesions occur around the knee, and less commonly, the humerus. Rarer locations include the small bones of hands and feet, scapula and pelvis [6]. These are usually benign and asymptomatic lesions, with malignant transformation reported in only 1 % [6]. Other reported complications include deformity, fracture, bursa formation and compression of adjacent nerves and vessels.
    Radiologic features include a well-defined, protuberant bony mass arising from the cortical surface of bone, typically arising in a metaphyseal/metadiaphyseal location of a long bone and pointing away from the joint. Lesions can be sessile or pedunculated; the pathognomonic feature being continuity of the medullary cavity with the underlying bone of origin [6, 7].
    On MR, these manifest as circumscribed, protruding lesions arising from the outer bony cortex. A peripheral zone of hypointense T1 and T2 signal surrounds a central zone of intermediate medullary fat signal. A cartilaginous cap is usually present in children and young adults, which shows an undulating junction with the underlying bony stalk. The MR features of the cartilaginous cap are those of well-differentiated hyaline cartilage, a low to intermediate signal on T1WI and a (markedly hyperintense) high signal on T2WI [8]. Increased malignant potential has been reported when the cartilaginous cap is > 2 cm thick (Fig. 3) [9].

**Fig. 3:**
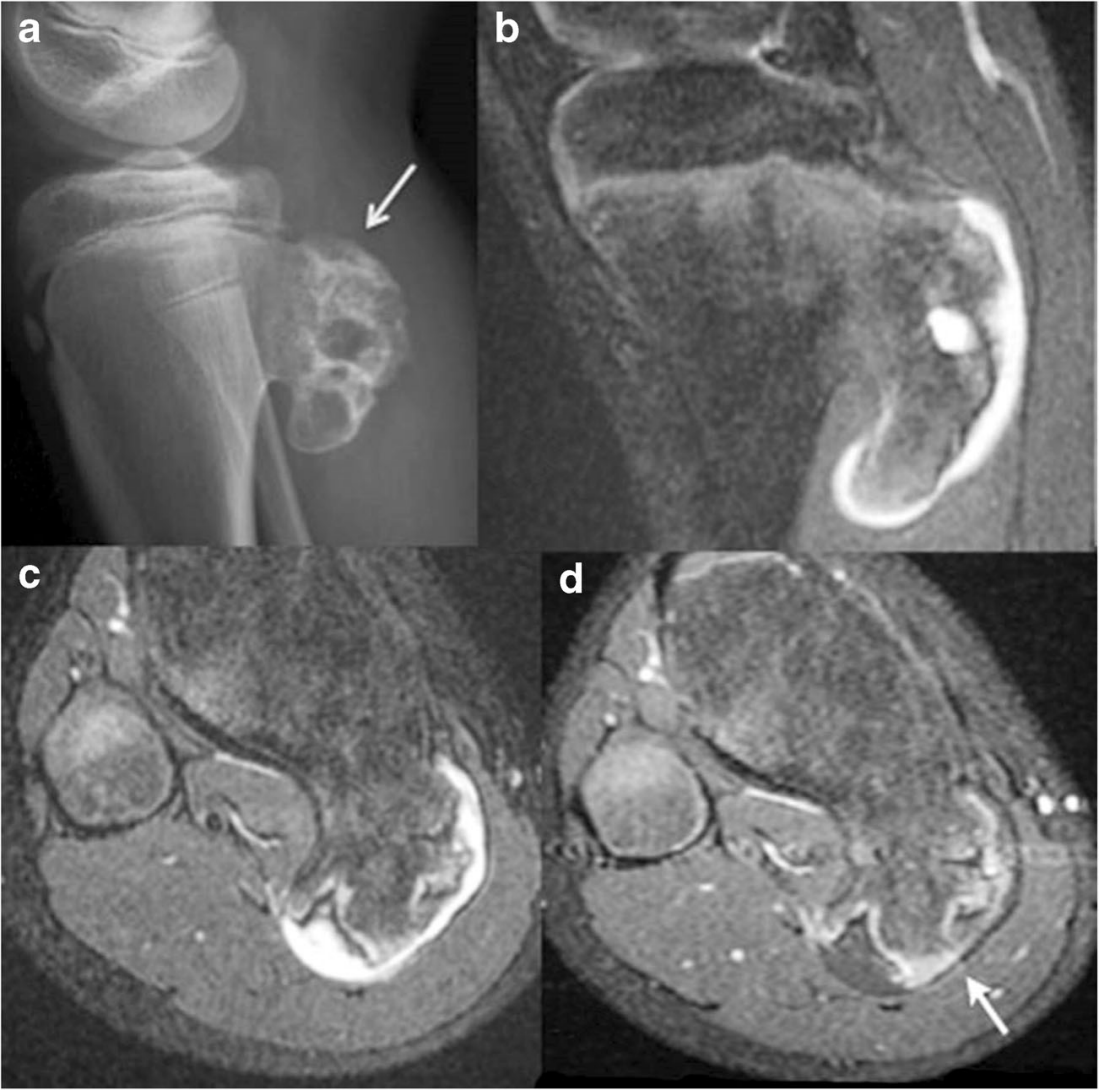
Benign solitary osteochondroma of the tibia in a 15-year-old boy. Lateral  radiograph of the knee **a** shows a bony protrusion arising off the posterior aspect of the proximal tibial metaphysis; note the continuity with the medullary cavity of the underlying tibia (*arrow*). Sagittal **b** and axial **c** T2 MR images show a markedly hyperintense, thin cartilage cap surrounding this bony exostosis; an axial T1 post contrast MR image **d** reveals a thin, peripherally enhancing cartilage cap (*arrow*)
    Subperiosteal osteoid osteoma
    Osteoid osteomas are benign bone-forming neoplasms, which account for approximately 13 % of benign bone tumours [10]. These occur mostly through the second and third decades of life. Broadly, these are classified based on location into medullary, intracortical and subperiosteal lesions. It is believed that many osteoid osteomas are, in fact, subperiosteal to begin with and later migrate into intracortical, endosteal or even medullary locations, secondary to continued bone remodeling, subperiosteal deposition and endosteal erosion [11].
    Radiographic findings include a circumscribed ovoid radiolucency or nidus measuring less than 1.5 cm, and surrounded by variable degrees of reactive bone formation and periosteal reaction (Fig. 4).

**Fig. 4:**
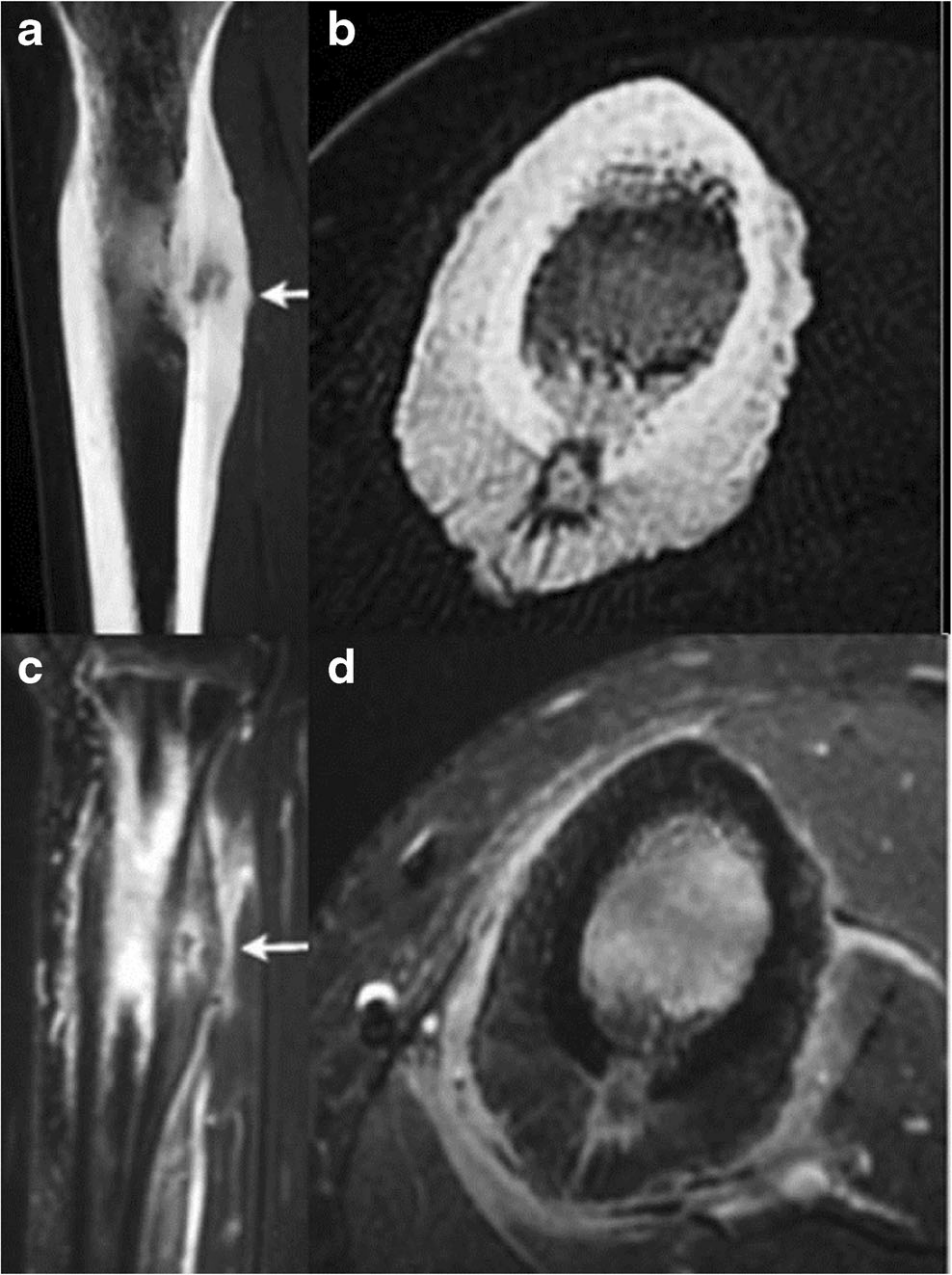
Sub-periosteal osteoid osteoma. A 15-year-old boy with sub-periosteal osteoid osteoma involving the right femur. Coronal **a** and axial **b** non-enhanced CT images show cortical thickening with reactive sclerosis (*arrow*) involving the postero-medial cortex of the femur, surrounding a low-attenuation nidus with central mineralization. Coronal STIR **c** and axial T1 post-contrast **d** images show avid contrast enhancement and edema within the bone marrow and soft tissue surrounding the nidus. The periphery of the nidus enhances more intensely than the central portion (*arrow*)
    On MR, there is typically dense fusiform thickening of the cortex, which has low signal on T1W, PDWI, T2WI, and fat-suppressed T2WI. Within the thickened cortex, there is a spheroid or ovoid zone (nidus) measuring less than 1.5 cm. The nidus can have low–intermediate signal on T1WI and PDWI, and low–intermediate or high signal on T2WI and FS T2WI. Calcifications in the nidus can be seen as low signal on T2WI. After gadolinium contrast administration, variable degrees of enhancement can be seen at the nidus [2].
    Fibrous cortical defect (FCD)
    FCDs are benign fibrohistiocytic lesions in the metaphyseal portions of long bones. The pathology is similar to non-ossifying fibromas (NOF), with differences primarily relating to size; fibrous cortical defects are small intracortical lesions, whereas the larger non-ossifying fibromas are located eccentrically in the medullary cavity. Up to 95 % of these lesions occur between the ages of 5 and 20 years [2].
    On radiographs, these present as lucent lesions with a sclerotic rim (Fig. 5). Cortical thinning or thickening, and bone expansion can be seen, more commonly with non-ossifying fibromas, given their larger size [2]. On fat-suppressed T2WI, a sclerotic rim is of low signal intensity is often seen. Variable degrees of gadolinium contrast enhancement are present.

**Fig. 5:**
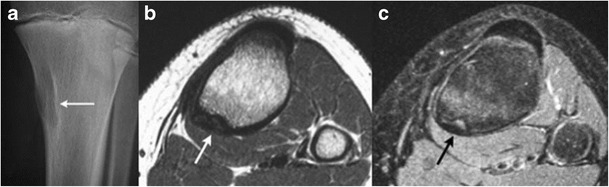
Fibrous cortical defect. A 13-year-old boy with a fibrous cortical defect in the proximal right postero-medial tibia. An antero-posterior radiograph of right tibia **a** shows an intra-cortical lucency with a thin rim of sclerosis (arrow). Axial T1 **b** and T2 fat-suppressed **c** images show a hypointense intracortical lesion with no medullary involvement. A hyperintense rim is seen on the T2-weighted image (*black arrow*)
  - Juxtacortical
    Juxtacortical/Periosteal chondroma
    These are benign protuberant hyaline cartilaginous tumours, which arise from the periosteum and are superficial to the bone cortex. Juxtacortical chondromas are rare lesions, accounting for < 1 % of overall bone lesions. More common in young adults, the exact incidence in childhood is unknown, although they have been reported in children [12]. The metaphyseal ends of long bones and small bones of the hands and feet are commonly involved.
    Radiologic features include a scalloped cortical surface with or without sclerosis, a thin periosteal shell and chondroid mineralization of the matrix [8]. On MR, typical signal characteristics of chondral tissue are noted, with an intermediate signal on T1WI and a heterogeneously hyperintense signal on T2WI [8]. Low-signal borders on T2WI and gradient recall echo (T2*WI) are often present, representing thin sclerotic reaction. Patchy matrix mineralization can contribute to interspersed areas of low signal on T2WI. Reactive perilesional or intramedullary oedema is not typical. Thin peripheral and septal contrast enhancement may be seen (Fig. 6) [8].

**Fig. 6:**
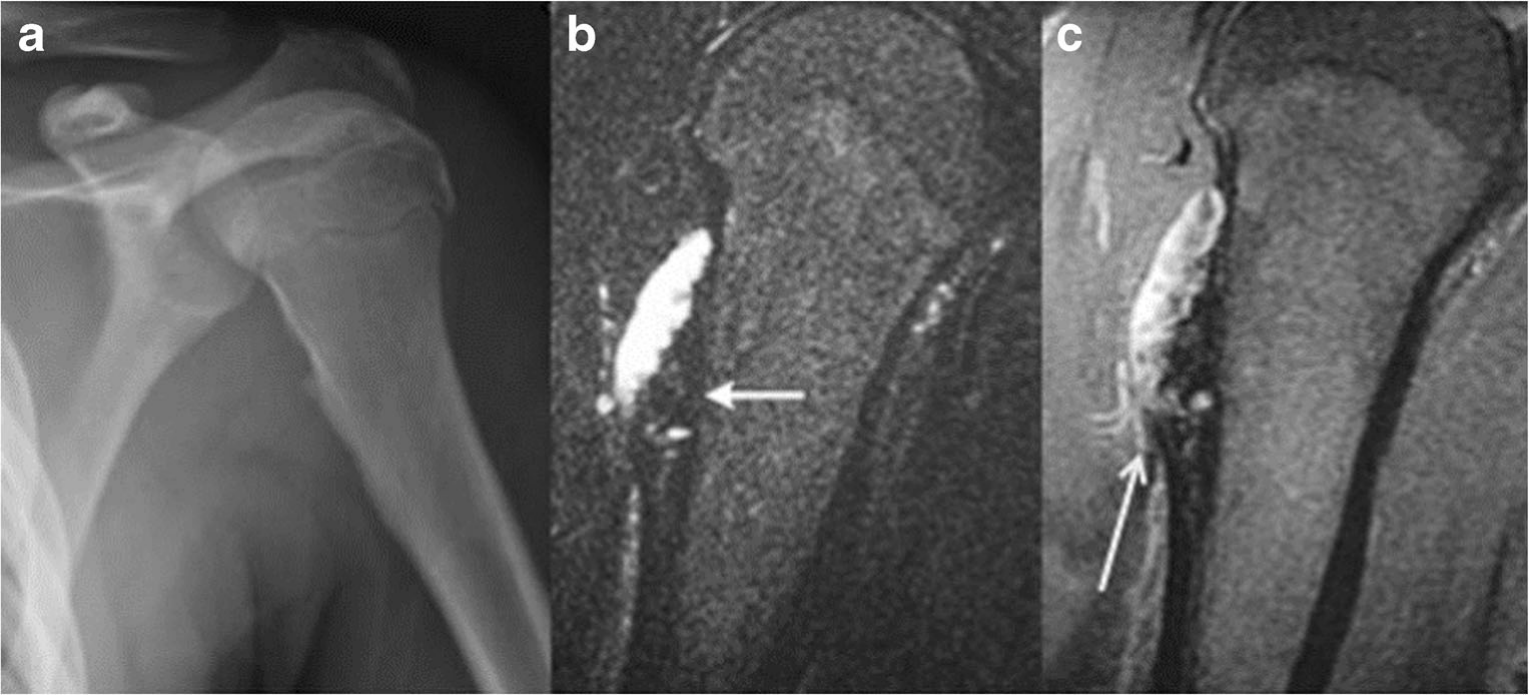
An 11-year-old boy with juxta-cortical chondroma at the proximal right humerus. A radiograph of the left shoulder reveals mild scalloping at the medial cortex of the proximal humeral metaphysis (*arrow*). A coronal T2 STIR image **b** and a coronal T1 post-contrast image **c** show a soft-tissue lesion abutting the cortex with a heterogeneously high T2 signal intensity suggesting cartilage (*thick arrow*), and predominantly peripheral contrast enhancement (*thin arrow*)
    Bizarre parosteal osteochondromatous proliferation (BPOP):
    BPOP, also known as Nora’s lesions, are rare, benign, reactive mesenchymal proliferations of bone adjacent to or attached to the outer periosteal surface of bone [13]. Proposed aetiologies include post-traumatic versus neoplastic. These commonly occur in the hands and feet (70 %), less commonly in long bones. Although mostly seen in adults, they are known to occur in children.
    These present radiologically as smooth and/or lobulated, variably calcified and/or ossified lesions, often with a broad base or stalk of attachment to an otherwise intact outer cortical surface of bone (Fig. 7). Lack of medullary continuity is the key differentiating feature from osteochondroma [14]. Remodelling of the adjacent bone cortex can occur. The lesion displaces but does not invade adjacent extraosseous soft tissues; adjacent soft-tissue oedema is seen with MR. High rates of recurrence have been documented post excision, in one study, up to 28 %[13].

**Fig. 7:**
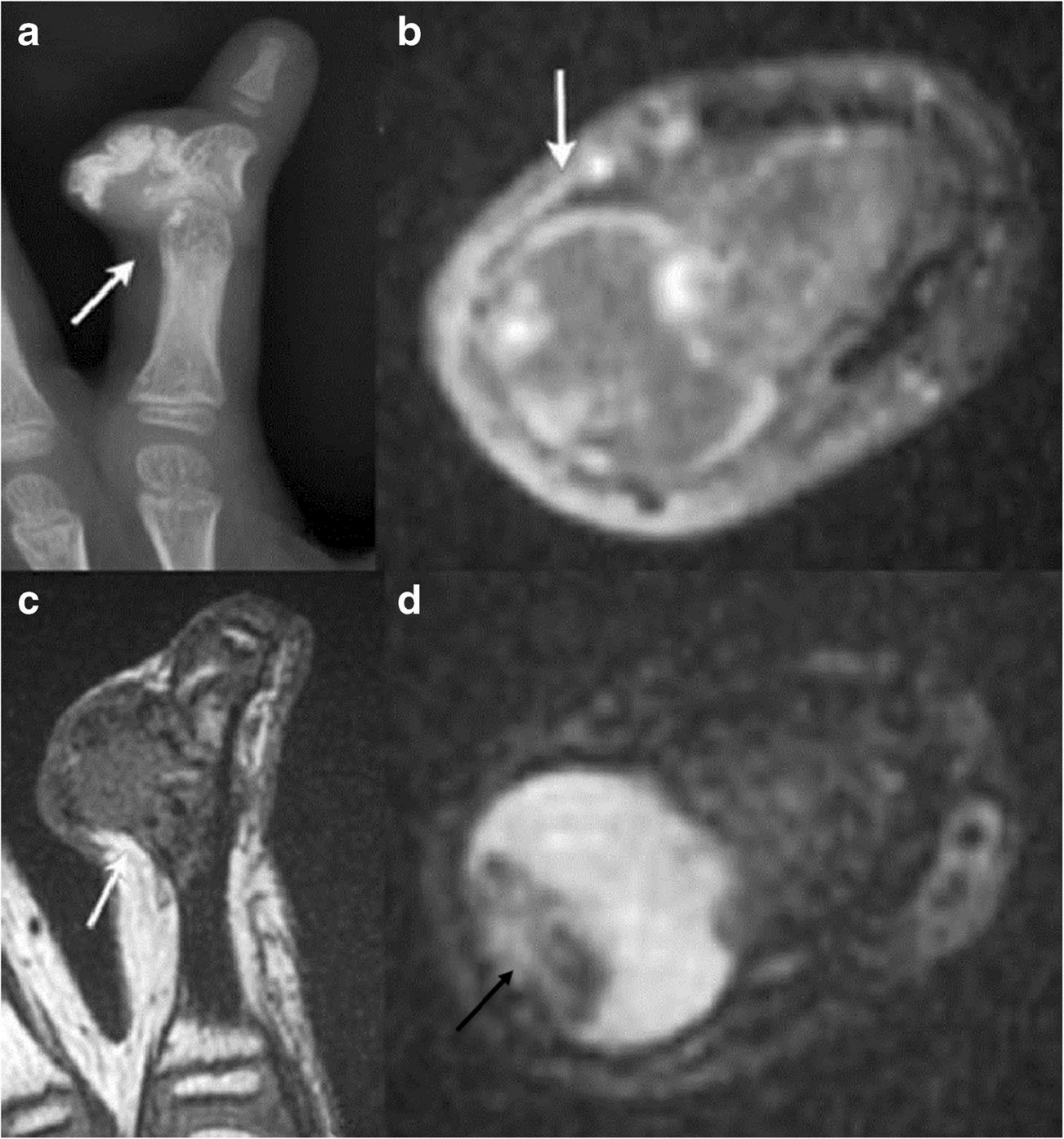
A 5-year-old boy with bizarre parosteal osteochondromatous proliferation at the middle phalanx of the right index finger. Antero-posterior radiograph of the right index finger **a** demonstrates a well-defined mass with amorphous calcifications (*arrow*) adjacent to the otherwise intact cortical surface of the middle phalanx. No medullary continuity is demonstrated between the lesion and underlying bone. Axial T1 post-contrast image **b** shows irregular peripheral contrast enhancement of the lesion without surrounding soft-tissue invasion (*arrow*). The lesion is hypointense on coronal T1 **c** and mostly hyperintense with few hypointense foci on axial T2 **d** (*black arrow*)
    Dysplasia epiphysealis hemimelica (DEH):
    This is also known as Trevor’s or Trevor–Fairbank disease and is a rare developmental disorder of the skeleton characterized by asymmetric osteochondral overgrowth of a medial or lateral epiphysis or epiphyseal equivalent [15]. This most frequently affects the knee and ankle, presents as a hard, painless mass adjacent to the affected joint with pain/discomfort developing at a later stage. The medial aspect of the epiphysis is more commonly affected than the lateral aspect.
    On radiographs, asymmetric cartilaginous overgrowth may be seen with multiple ossific centres and a stippled, irregular or dense pattern of epiphyseal chondral calcification. Affected epiphysis is usually larger than that of the contralateral normal limb. Associated metaphyseal widening and remodelling may occur [15].
    MR is the modality of choice for identifying dimensions of the cartilage mass, neurovascular or ligamentous involvement, associated complications such as bursitis and arthritis and distinction from other pathology. The typical imaging characteristics of cartilage are seen on both noncontrast and post-contrast MR. With increasing lesion maturity, MRI demonstrates cortical and medullary coalescence between the ossification centre of the lesion and the adjacent epiphysis — the marrow signal intensity parallels that of the normal bone (Fig. 8). Signal voids on T1/T2 represent foci of dense calcification within the marrow [15].

**Fig. 8:**
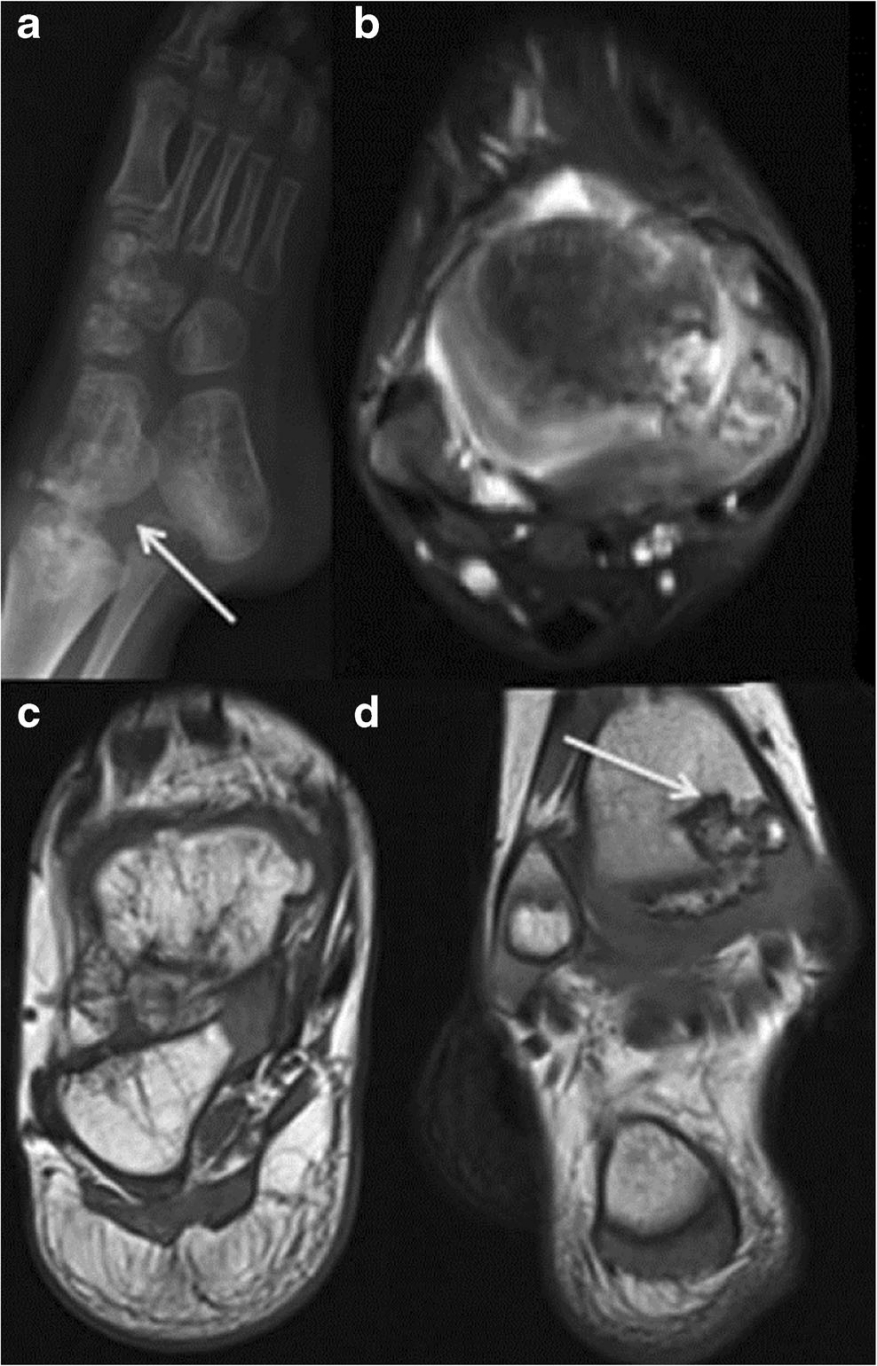
Trevor’s disease (dysplasia epiphysealis hemimelica) in 3-year-old boy. Antero-posterior radiograph of the left foot **a** shows asymmetric osteochondral overgrowth arising from the medial epiphysis of the tibia (*arrow*) and from multiple ossification centers within the tarsal bones of the foot. An axial STIR image **b** and axial and coronal T1 images **c** and **d** demonstrate cortico-medullary coalescence and cartilaginous signal (*arrow*)
    Heterotopic ossification
    These represent localized, non-neoplastic, reparative lesions in soft tissues separate or adjacent to bone, which are comprised of reactive hypercellular fibrous tissue, cartilage, and/or bone. They can arise secondary to trauma (*myositis ossificans circumscripta*, *ossifying hematoma*), or without a history of prior injury.
    On MR, variable low signals on T1WI, PDWI, and T2WI are noted, depending on the degree of mineralization/ossification, fibrosis, and hemosiderin deposition (Fig. 9). Zones of high signal on T1WI and T2WI may occur from fatty marrow metaplasia. Enhancement with gadolinium contrast or associated abnormal marrow signal are usually absent [2]

**Fig. 9:**
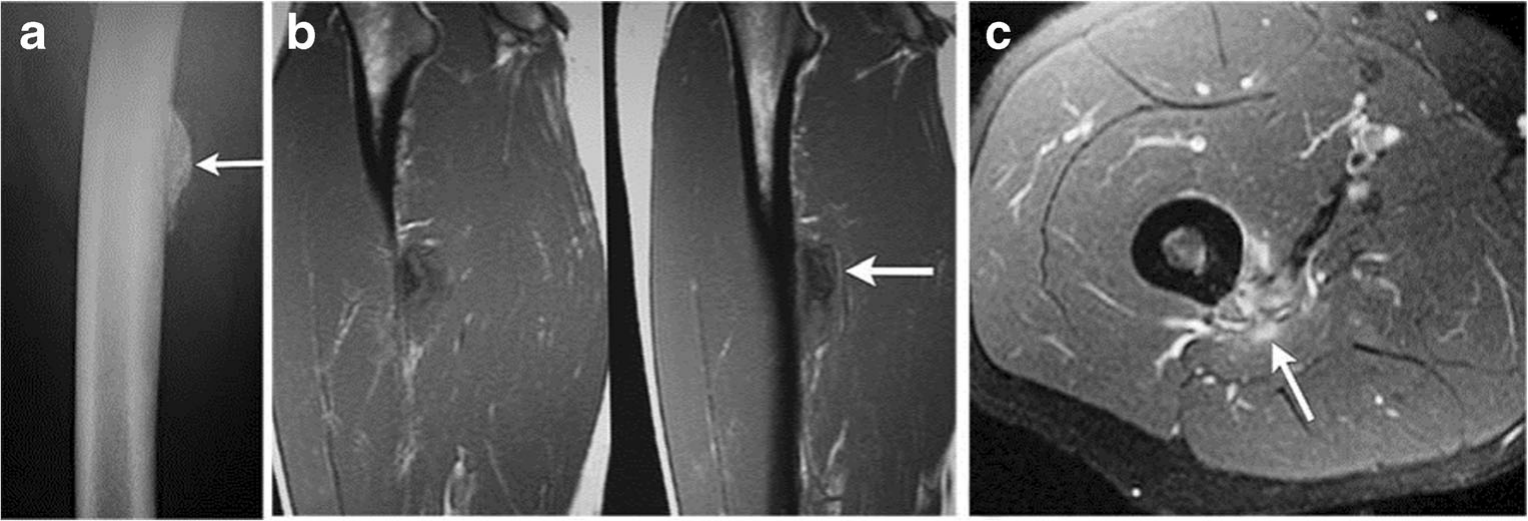
Heterotopic ossification in an 18-year-old boy. Lateral radiograph of the femur **a** shows an ossifying lesion adjacent to but distinct from the lateral aspect of the proximal femoral cortex (*arrow*). Sagittal T1-weighted MRI **b** demonstrates heterogeneously low signal through this region. Axial T1-weighted post-contrast image obtained at this level **c** shows associated enhancement (*arrow*). There was a history of trauma
  - Periarticular
    Ganglion cyst:
    Ganglia are juxta-articular benign cystic lesions, rich in hyaluronic acid and other mucopolysaccharides [16]. They are believed to arise secondary to myxoid degeneration of peri-articular connective tissue as sequelae of prior trauma, or inflammation [17]. Ganglia may be intra- or extra-articular, periosteal or intra-osseous, and do not communicate with the joint as often as synovial cysts [16]. Lack of a true endothelial lining constitutes the differentiating feature from synovial cysts [18]. Ganglion cysts can arise from the joint capsule, ligaments, tendon sheaths, bursae or subchondral bone [19].
    On MR, these are sharply defined fluid collections with low to isointense signals on T1-weighted images, and a homogeneous high signal on T2, proton density and STIR-weighted images; the degree of this hyperintensity inversely correlates with the protein content of the fluid (Fig. 10) [19].

**Fig. 10:**
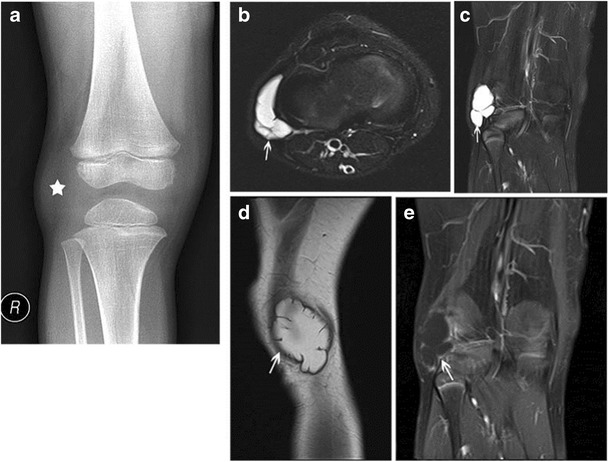
Ganglion cyst at the lateral margin of the right knee in a 6-year-old boy. An AP radiograph of the right knee **a** reveals slight soft-tissue fullness along the lateral aspect of the knee (*marked by star*). This corresponded to the site of fluctuant mass noted on palpation. No associated adjacent bony defects/erosive changes are observed. Axial **b** and coronal **c** fat-suppressed T2-weighted MR images show a lobulated, deformable, cystic mass adjacent to and wrapping around the lateral distal femoral metaphysis (*arrows*). A sagittal proton density image **d** reveals high signal within the fluid (due to high mucopolysaccharide content); thin rim enhancement was noted with contrast (*arrow*) **e**
    Peripheral rim-like gadolinium contrast enhancement can be seen, or there can be complete lack of enhancement. Usually, no solid/enhancing components are seen; although, occasionally, internal T2 hypointense debris or even osseous loose bodies can be seen [19]. Erosion/invasion of adjacent bone may be present.
    Haemorrhage or infection may occur within these lesions, resulting in wall-thickening and a heterogeneous internal signal on MR. Adjacent oedema and fluid tracking can be seen with cyst rupture [19].
    b. Post-traumatic
      Chronic avulsive injuries/cortical desmoid:
        Cortical desmoids are usually a consequence of an avulsive injury or stress reaction at the insertion site of the medial head of the gastrocnemius muscle or adductor magnus.
        On plain films, these appear as small radiololucent saucer-shaped lesions or areas of cortical irregularity with an associated sclerotic base, typically at the posteromedial aspect of the distal femoral condyle (Fig. 11).

**Fig. 11:**
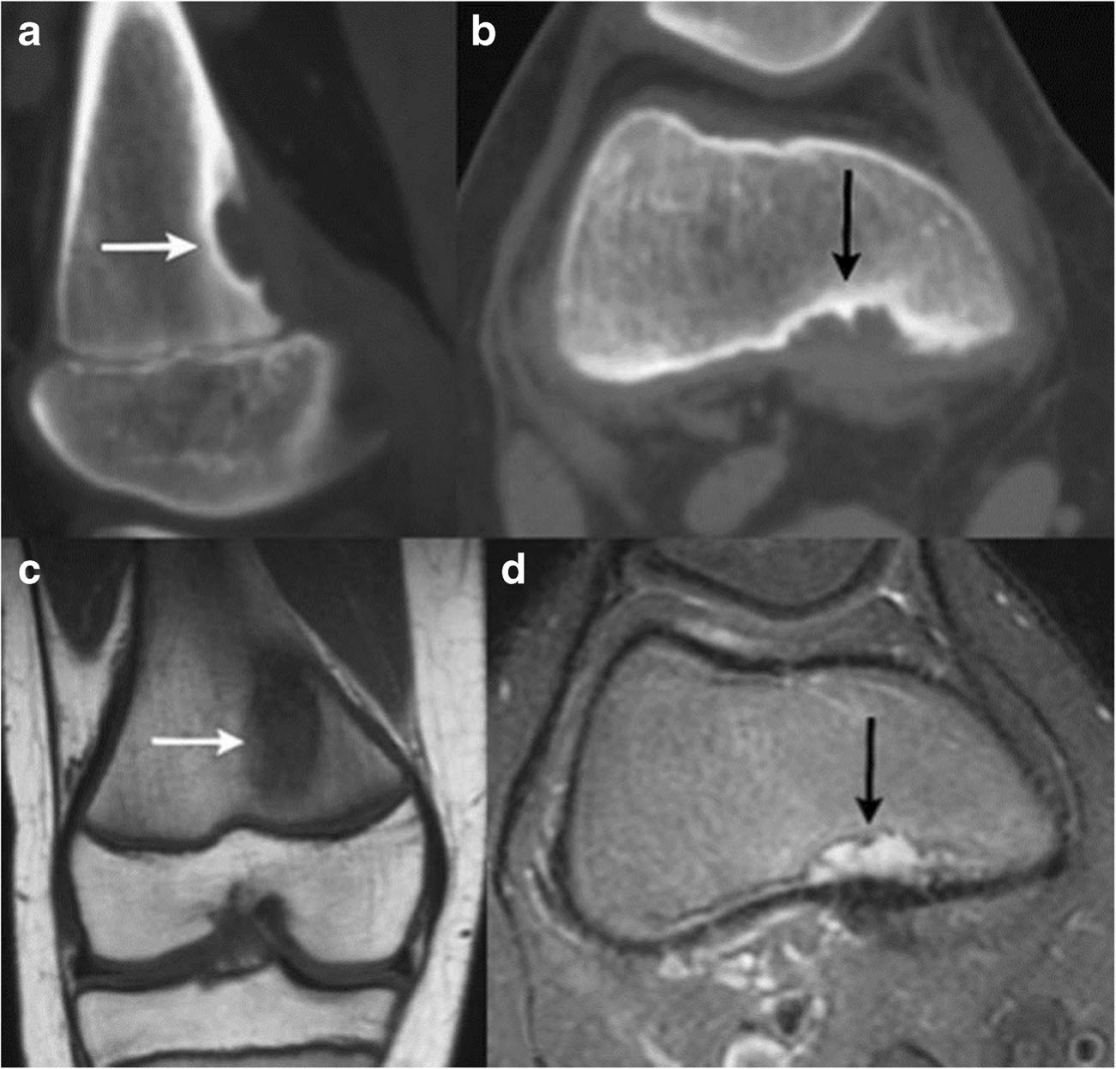
Cortical desmoid at the postero-medial aspect of the left femoral metaphysis in a 10-year-old girl. Sagittal **a** and axial **b** CT images show a scalloped radiolucent lesion with a thin sclerotic rim (*arrow*) along the posteromedial distal femoral metaphysis. Coronal T1 **c** and axial T2 fat-suppressed **d** images demonstrate low signal on T1 and high signal on T2 (*white arrows* on **c** and *black arrows* on **d)**
        On MR, these lesions usually have a low to intermediate signal on T1WI and PDWI, intermediate to slightly high signal on T2WI, and high signal on FS T2WI. A thin border of low signal on T1WI and T2WI is often seen at the inner margin of the lesion corresponding to a thin zone of sclerosis seen on plain films and/or CT. Bone marrow deep and peripheral to the lesion may have a slightly high signal on FS T2WI. The lesion itself and the surrounding marrow may enhance with gadolinium [2].
    c. Infectious/Inflammatory
      Certain unique categories of infectious/inflammatory processes of bone qualify as “surface lesions” due to their location. These include septic cortical osteitis, intracortical Brodie’s abscesses and the occasional periostitis or isolated cortical involvement seen with chronic recurrent multifocal osteomyelitis. All these entities can manifest with periosteal reaction and/or cortical disruption at the periosteal surface of the cortex.
  -Septic cortical osteitis:
    Bone infection, commonly accompanied by adjacent soft-tissue infection, usually occurs secondary to hematogenous spread, direct inoculation or spread from adjacent tissues [2].
    Septic cortical osteitis is a rare but distinct type of bone infection characterized by a hematogenously seeded infection predominantly or exclusively limited to the bony cortex. This is most commonly bacterial in aetiology (Staph. Aureus is the commonest implicated organism).
    Typical imaging features include vertically oriented cortical osteolysis described as the “cortical split sign” and disruption of the bony cortex at the periosteal side [20].
    MR findings of bone infection are characterized by a low signal on T1WI and PDWI, and high signal on T2WI and FS T2WI. There is loss of definition of the cortical line, and the involved portions of the bone enhance with gadolinium contrast (Fig. 12). Associated soft-tissue swelling, with or without focal abscess formation (Fig. 13), can have a similar high signal on T2WI and Gd contrast enhancement on FS T1WI. Subperiosteal fluid collections typically have low signal on T1WI and high signal on T2WI, FS T2WI and STIR [2].

**Fig. 12:**
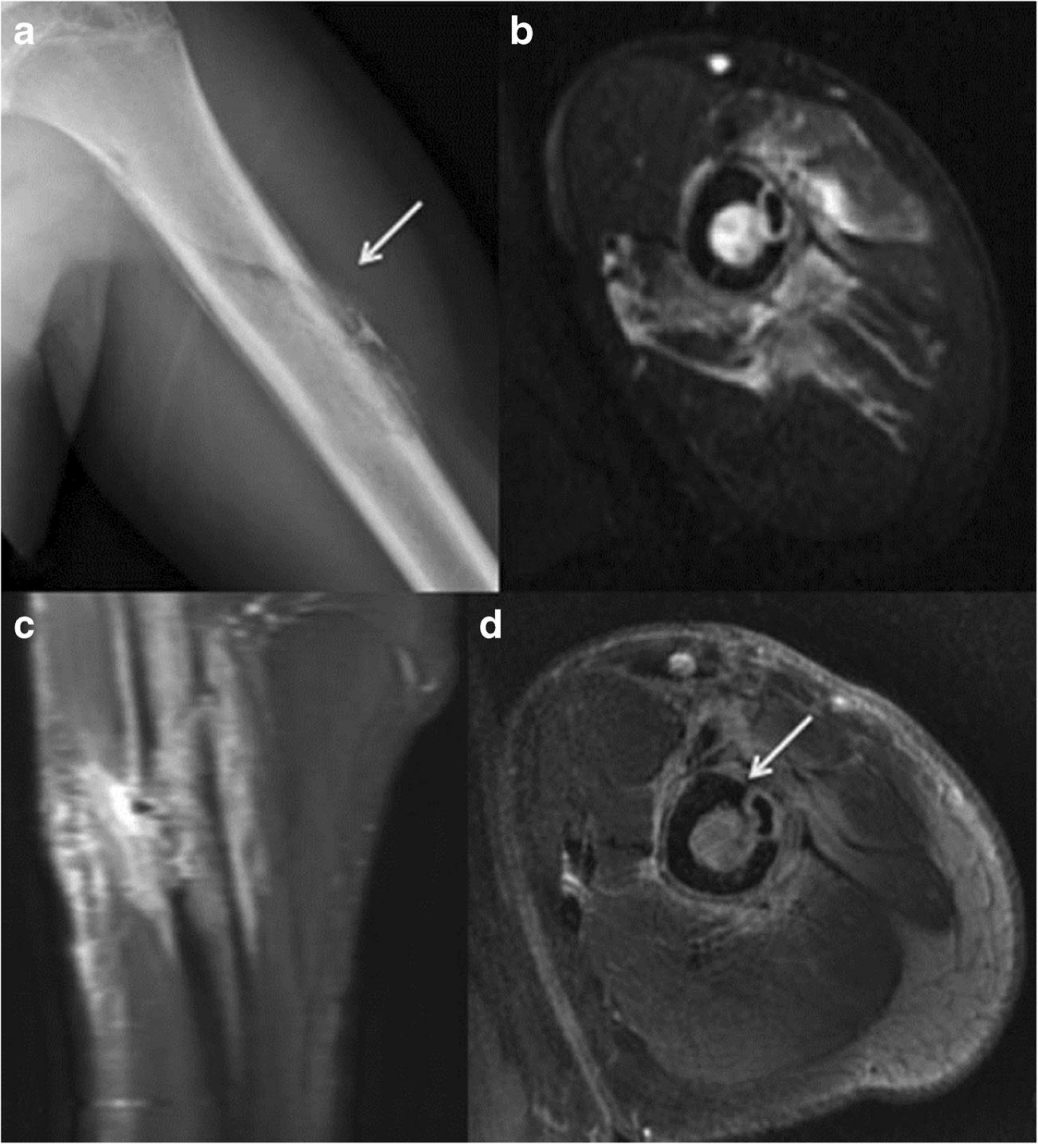
Septic cortical osteitis. Antero-posterior radiograph of the left humerus **a** shows irregular inhomogenous lamellated periosteal reaction at the left lateral cortical surface of the diaphysis (*thick arrow*). There is an associated obliquely oriented fracture through the proximal humerus. Axial fat suppressed T2 **b** and coronal fat suppressed T2 **c** images demonstrate an intracortical hypointensity with surrounding edema through the medullary cavity and the adjacent soft tissues. An axial post-contrast injection image **d** shows peripheral contrast enhancement surrounding an intracortical hypointensity and enhancement through the medullary cavity and surrounding soft tissues (*thin arrow*)

**Fig. 13:**
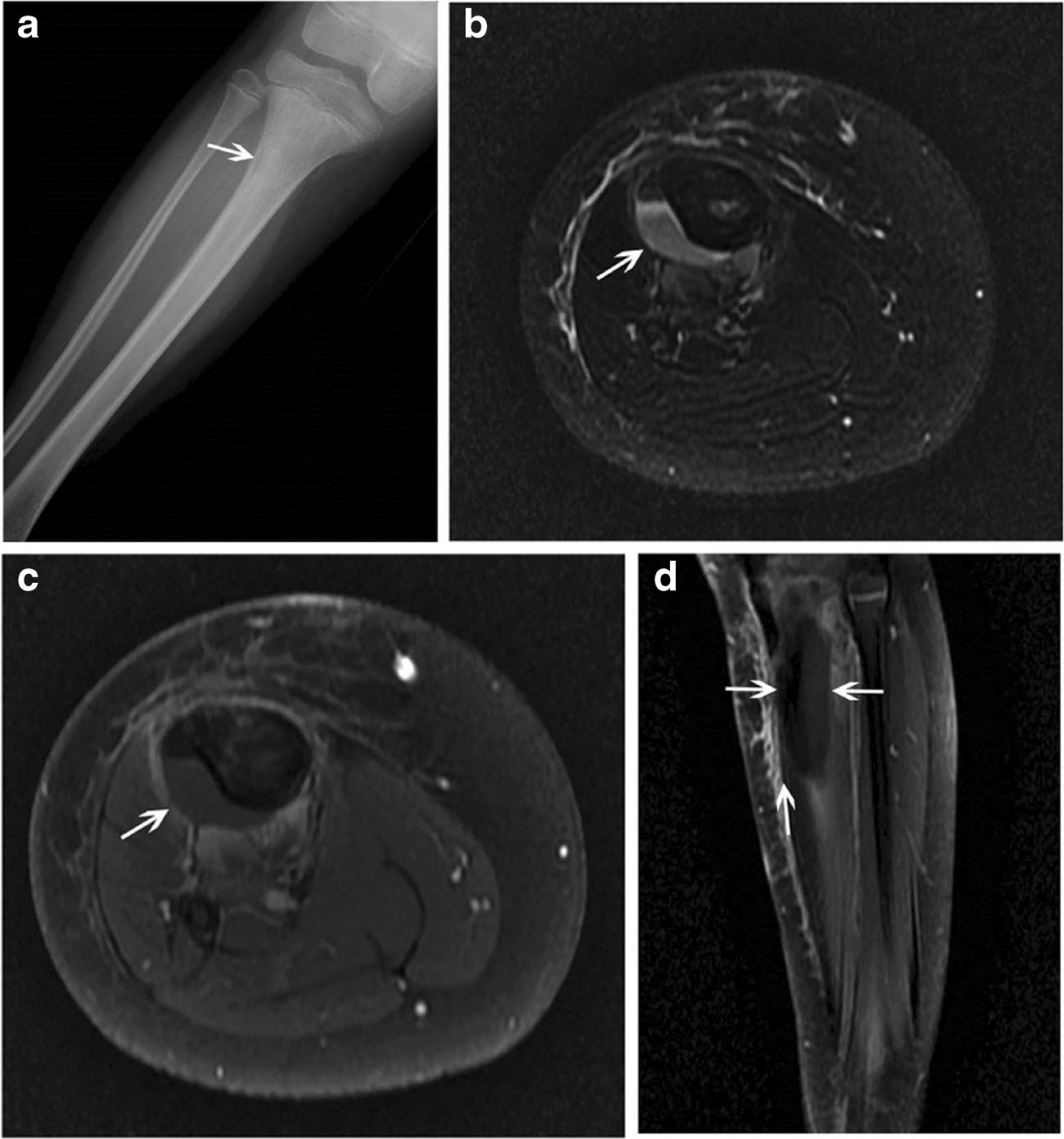
Septic cortical osteitis with subperiosteal abscess formation in a 7-year-old girl. Antero-posterior radiograph of the left tibia **a** shows an ill-defined lucency at the lateral aspect of the proximal tibial metaphysis (arrow). Axial  STIR image **b** reveals a crescentic, hyperintense fluid collection adjacent to the proximal tibia at this level. This collection is hypointense with peripheral enhancement as seen on the post-contrast axial **c** and sagittal **d** images. Enhancement can also be seen through surrounding soft tissues
    During the subacute phase of an evolving bony infection, the transition zone between normal and abnormal bone becomes sharper as the process becomes more localized [21].
  -Intracortical Brodie’s abscess:
    Brodie’s abscess is a manifestation of subacute osteomyelitis, and though usually metaphyseal/medullary in location, can rarely be intracortical. When intracortical in location, it can present radiographically as an irregular lytic abscess cavity within the bony cortex with associated solid periosteal reaction [1].
  -Chronic recurrent multifocal osteomyelitis (CRMO):
    CRMO is a non-bacterial, auto-inflammatory disease characterized by recurrent flares of inflammatory bone pain related to presence of multiple foci of aseptic osteomyelitis [22]. Children and adolescents comprise the main affected demographic with the mean age at manifestation determined to be 11.4 years on a German incidence surveillance study (Fig. 14) [23].

**Fig. 14:**
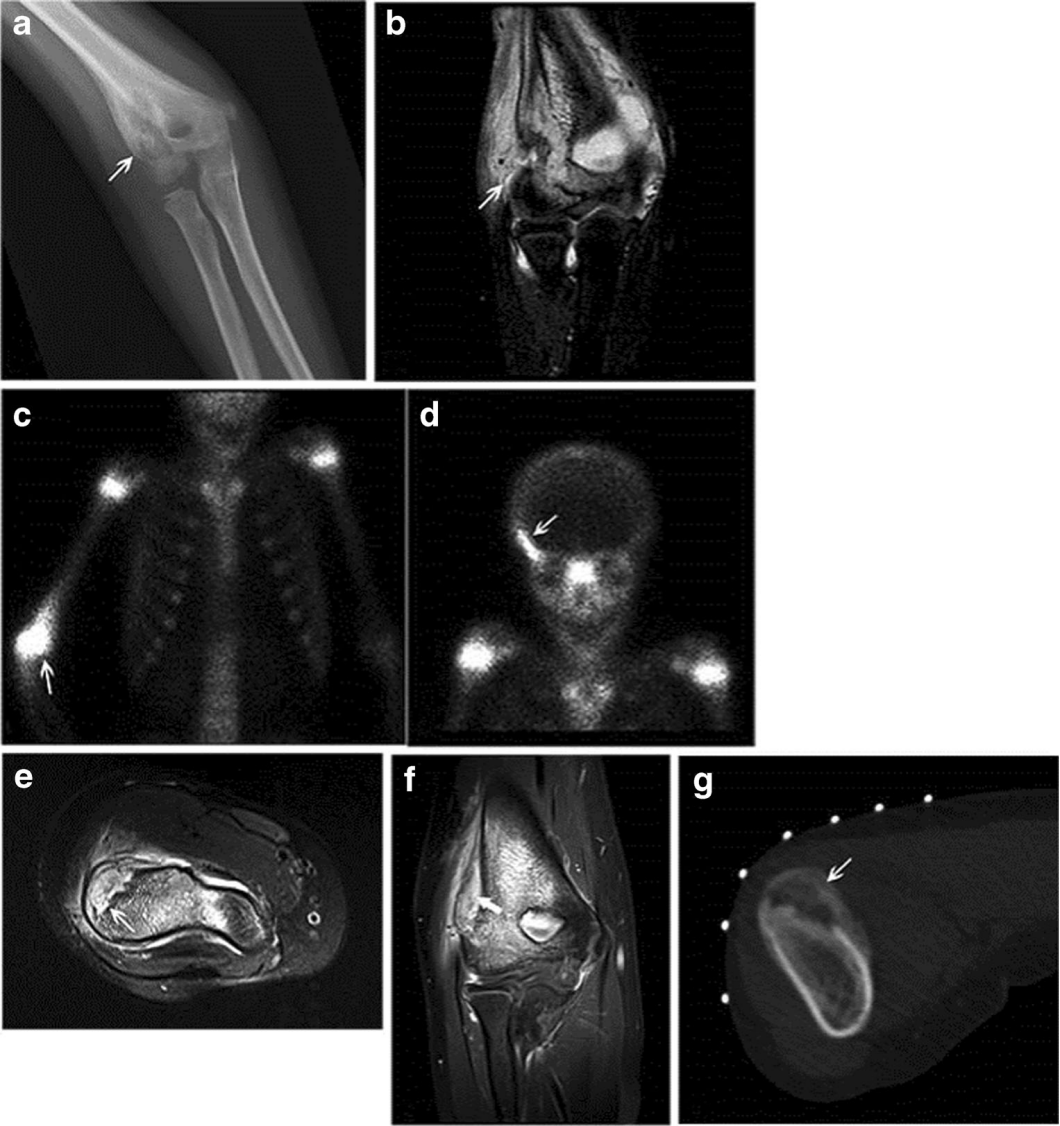
Chronic recurrent multifocal osteomyelitis (CRMO) in an 8-year-old girl. An anteroposterior radiograph of the right elbow **a** reveals an eccentric, mostly lucent lesion at the lateral distal humerus (*arrow*) with surrounding periosteal reaction. Corresponding coronal T2 STIR image **b** reveals hyperintense, abnormal signal eccentrically along the distal lateral humerus with overlying periosteal reaction and surrounding soft-tissue edema. Tc-99m-labeled MDP bone scan reveals increased radiotracer uptake at the distal right humerus **c** and the right sphenoid **d**, suggesting multifocality of the process. The patient had a chronic course with recurrent exacerbations. Axial STIR **e** and coronal T1 post-contrast **f** MR images obtained 2 years after the initial presentation reveal STIR hyperintense, enhancing abnormality along the lateral distal humerus (*thin arrows* on **e**, *thick arrow* on **f**). The abnormality is peripheral, and involves the cortex and overlying periosteum. A non-contrast axial CT image **g** obtained at the same time for biopsy guidance shows cortical irregularity through the affected portion of the lateral distal humerus with significant overlying periosteal reaction (*arrow*)
    On plain radiographs, lesions are characteristically juxtaphyseal, osteolytic, sclerotic or mixed and lack periosteal reaction [22]. Less commonly, some patients, particularly those with unifocal lesions, show breached cortices and periosteal appositions, in which case distinction from the tumour becomes important [24]. Rarely, diaphyseal lesions with only cortical thickening have been reported [25]. Such periosteal/isolated cortical involvement, though overall uncommon in the setting of CRMO, falls under the category of lesions involving the outer cortical surface of the bone, which are being addressed in this review.
    Multiple lesions, if not visible radiographically can be detected using radionuclide bone scanning or whole-body MRI — the latter being the more sensitive method of evaluation [25]. Lesions are typically hypointense on T1 and hyperintense on T2 [25]; MR is also useful for detection of synovial enhancement near the bone lesions [26].
2. Malignant:
  -Primary surface bone malignancies
    Osteosarcomas are malignant connective tissue tumours comprised of proliferating neoplastic spindle cells which produce an osteoid matrix, cartilage matrix and fibrous tissue.
    Based on WHO definitions, juxta-cortical osteosarcomas include all surface osteosarcomas-parosteal, periosteal and high-grade surface osteosarcoma.
    Periosteal osteosarcomas arise from the inner, germinative periosteal layer (Fig. 15) whereas parosteal osteosarcomas arise from the outer, fibrous layer of the periosteum (Fig 14). High-grade surface osteosarcomas arise directly from the bone surface and have the worst prognosis of all the surface osteosarcomas.

**Fig. 15:**
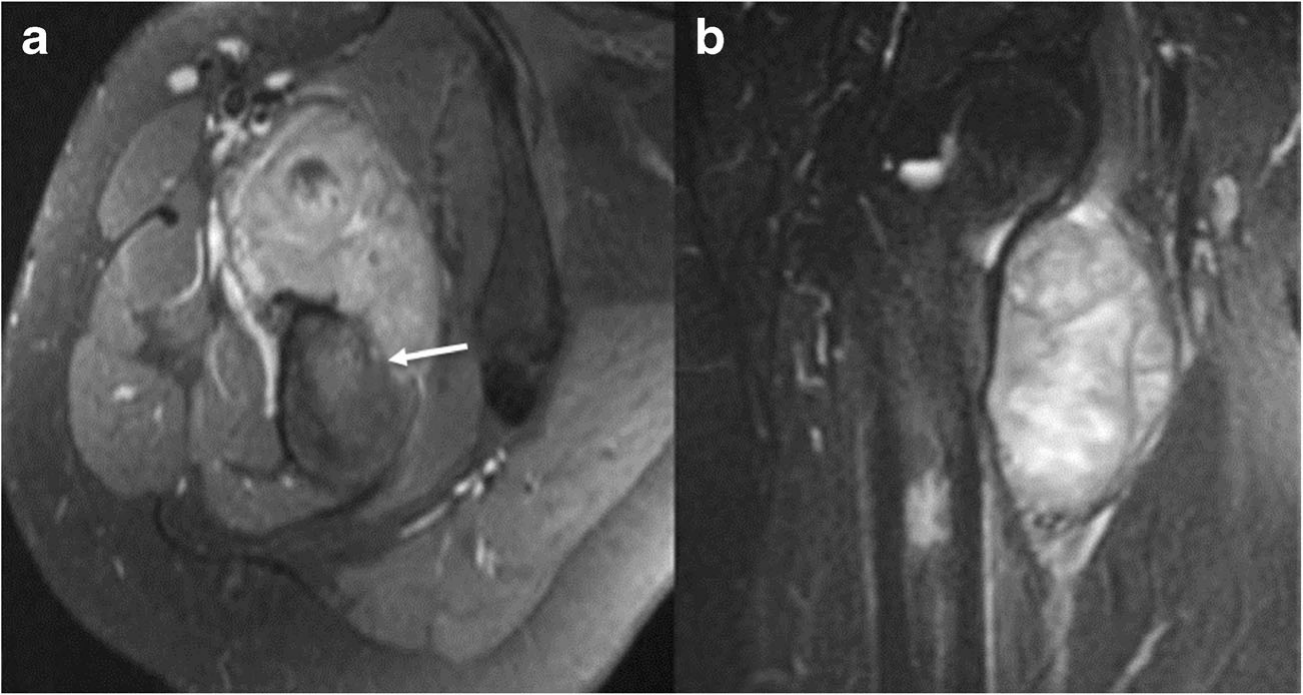
Periosteal osteosarcoma in the right proximal femur. Axial **a** and coronal **b** T2 images reveal high signal soft-tissue mass involving predominantly the vastus medialis and intermedius with cortical disruption and intraosseous extension at the anteromedial aspect of the right femur (*arrow*)
    Parosteal osteosarcomas arise at the metaphyses of tubular bones; periosteal lesions are predominantly diaphyseal whereas high-grade surface tumours may be either [27].
    On MR, the mineralized portions of these tumours usually have a low signal on T1WI, PDWI, T2WI, FS PDWI and FS T2WI (Fig. 16). The soft-tissue portions of these tumours often have a low–intermediate signal on T1WI, an intermediate to slightly high or high signal on T2WI, and a high signal on FS PDWI and FS T2WI. Areas of haemorrhage, cystic change and/or necrosis with or without associated fluid-fluid levels may be present. Non-necrotic or non-mineralized soft-tissue portions of these tumours usually show Gd contrast enhancement as well as sites of invasion into the medullary space and adjacent soft tissues [2].

**Fig. 16:**
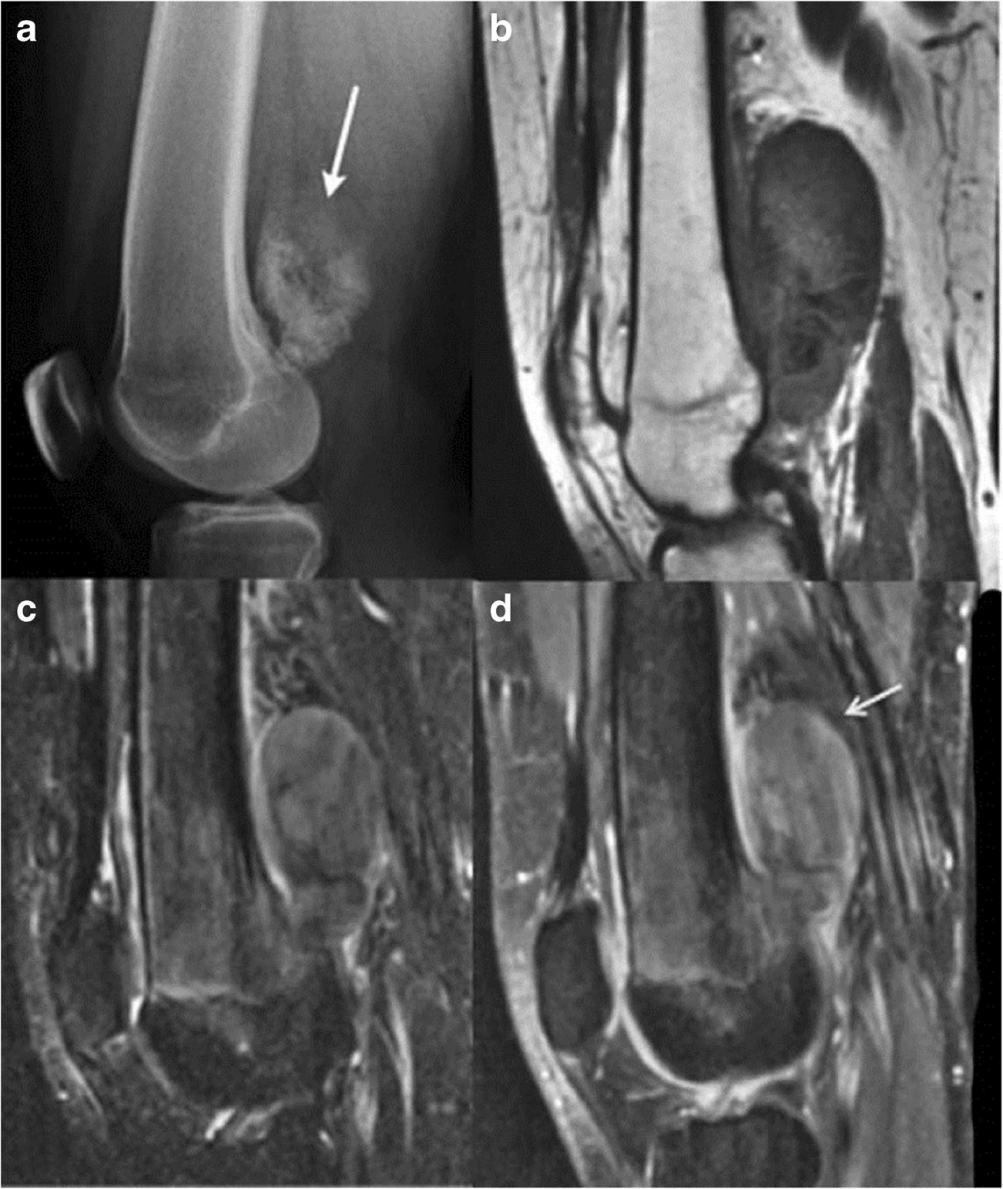
Parosteal osteosarcoma on the left distal posterior femur. A lateral radiograph of the left knee **a** shows a large exophytic mass with central dense ossifications adjacent to the femur (*arrow*). Sagittal T1 **b** and T2 **c** MR images demonstrate heterogeneous signal through this large soft-tissue tumor on both T1 and T2. A sagittal T1 post-contrast image **d** reveals heterogeneous contrast enhancement (*arrow*)
3. Periosteal reaction/abnormalities:
  Periosteum is a bi-layered zone, comprising of an outer fibrous layer and an inner cambium; the latter is dynamic and has osteoblastic potential. Periosteal new bone formation can occur during periods of normal growth, in response to injury, or in association with numerous benign, malignant and systemic conditions. Periosteal activity is more pronounced in children than in adults, and periosteal elevation occurs more easily in children [21].
  Based on radiographic appearances, the broad categories of periosteal reaction include lamellated, solid, spiculated, and Codman’s triangle. All these types can be continuous or interrupted.
  -Regional periosteal reaction:
    Non-interrupted periosteal reaction from stress fractures and healing traumatic fractures manifests as a linear thin single 1–2-mm band of low signal superficial to the bone cortex (Fig. 17). Tissue deep to the periosteum has an intermediate signal on T1WI, a slightly high to high signal on T2WI, and enhances with gadolinium contrast [2]. Single-layered periosteal reaction can also be physiologic in preterm infants up to 6 months of age.

**Fig. 17:**
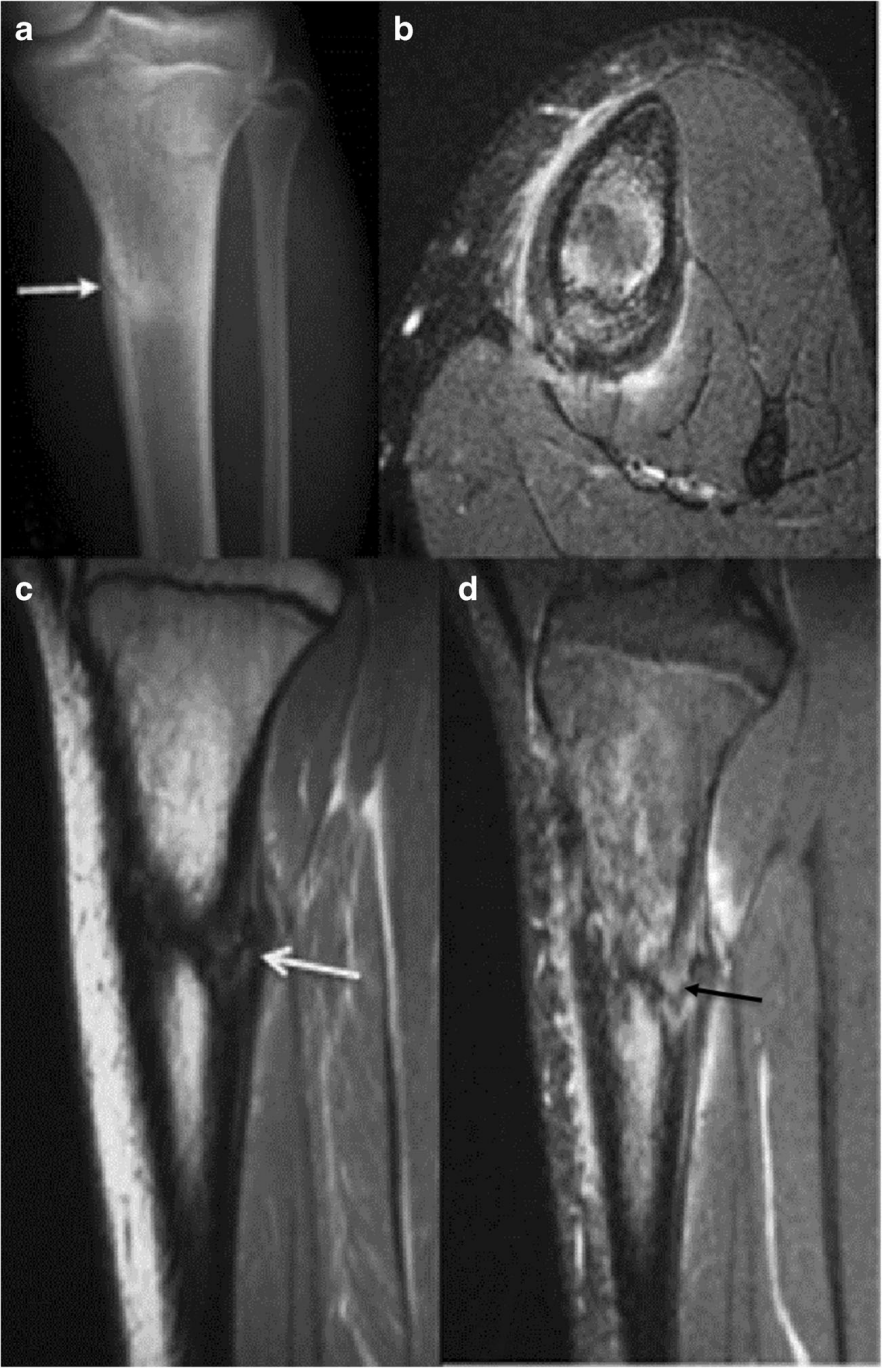
Periosteal reaction post trauma. An antero-posterior radiograph of the leg bones **a** shows a healing fracture at the postero-medial aspect of the tibia. Adjacent cortical thickening and surrounding sclerosis are seen (*arrow*). Axial T2 **b** coronal T1 **c** and coronal T2 **d** images demonstrate a hypointense healing fracture line with the surrounding bone and soft-tissue edema
    Multilamellated/“onion skin” periosteal reaction is seen most commonly with aggressive tumours such as Ewing’s sarcoma and osteosarcoma, and can also be seen with histiocytosis.
    Solid periosteal new bone is the hallmark of benign, slow, persistent lesions such as osteoid osteomas. Periosteal reaction associated with osteomyelitis and Langerhans’s cell histiocytosis may be either be single-layered, multilamellated or solid [21].
    Spiculated periosteal reaction can present as “hair-on-end”, divergent or sunburst patterns and generally occurs with more rapidly unfolding processes such as aggressive malignancies including osteosarcoma, Ewing’s sarcoma or neuroblastoma.
    Codman’s triangle is an interrupted type of periosteal reaction, which is accompanied by a cuff of reactive bone at the extreme ends of a lesion [21].
  -Diffuse periosteal reaction:
    A discussion about periosteal pathology is incomplete without a brief overview of some systemic conditions, which cause widespread periosteal reaction.
    Infantile cortical hyperostosis (Caffey’s disease) is a self-limiting inflammatory disorder of unknown aetiology affecting infants less than 5 months [28]. Common findings include soft-tissue swelling (frequently over the mandible), fever, hyperirritability, and cortical hyperostosis (ribs, ulna, tibia, fibula, humerus, femur, radius, metacarpal and metatarsal bones). Imaging findings on radiographs and MR include smooth, wavy, and/or irregular diaphyseal or metaphyseal periosteal and cortical thickening affecting one or multiple bones.
    Diffuse, symmetric periostitis may also occur as a complication of long-term prostaglandin therapy which is administered to maintain ductus arteriosus patency in infants with congenital heart disease (Fig. 18) [29]. Such periostitis is associated with extremity swelling and improves on cessation of prostaglandin infusion [30].

**Fig. 18:**
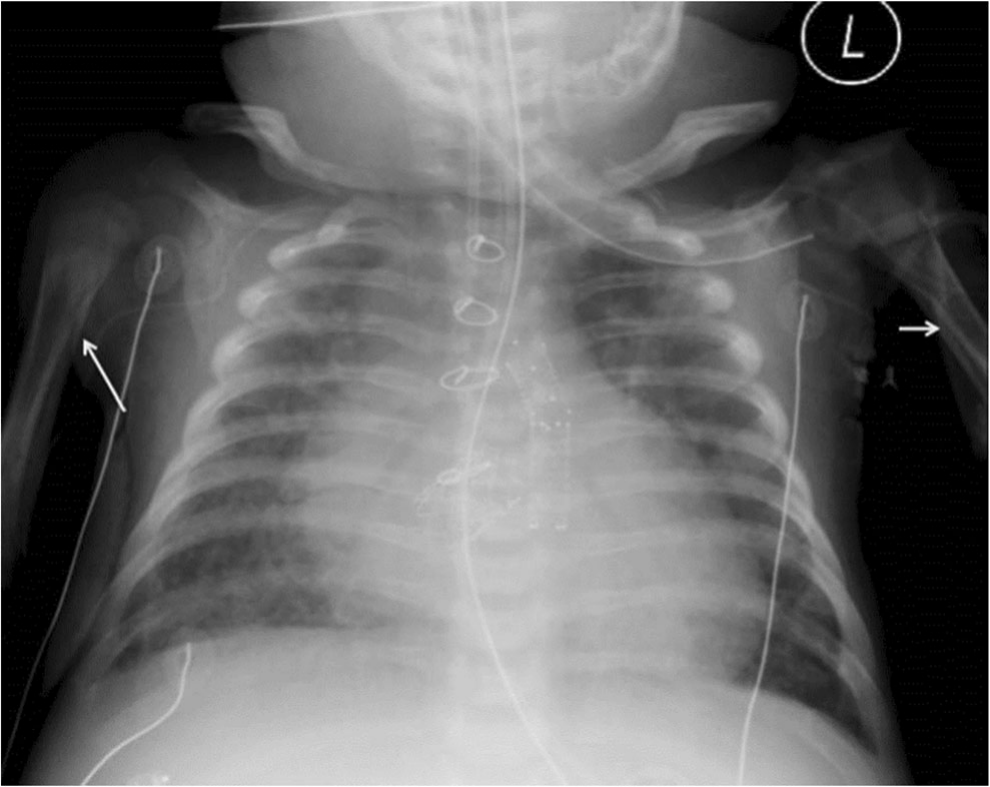
Diffuse periosteal reaction from postprostaglandin therapy in a 3-month-old infant. An antero-posterior radiograph of the chest shows diffuse periosteal reaction at the medial aspects of both humeri (*arrows*), along the undersurface of the clavicles and surrounding the ribs. This patient has a complex congenital heart disease for which he received cardiac surgery including a patent ductus arteriosus (PDA) stent
    Hypervitaminosis A in children affects one or multiple bones (ulna, metatarsal bones, clavicle, tibia, fibula, other bones). Physeal damage results in metaphyseal deformity and premature physeal fusion.
    Hypovitaminosis C/scurvy is uncommon. Groups at risk include infants who are fed evaporated or boiled milk, in which ascorbic acid is destroyed by heat, and dietary restrictions stemming from psychiatric or developmental disorders [31]. In young children, vitamin C deficiency after 4 to 10 months can result in infantile scurvy with clinical features including failure to thrive, petechial hemorrhages, ulcerated gingiva, hematemesis, melena, hematuria, and anaemia. On radiographs, periosteal elevation with new bone formation can be seen in the diaphyseal and metaphyseal portions of one or multiple bones from sub-periosteal hemorrhage. Metaphyseal beak-like bone protrusions can occur from the abnormal zones of provisional calcification at the physeal plate, as well as sub-epiphyseal fracture.

## Conclusions

To conclude, paediatric bone surface lesions comprise a broad spectrum of pathology ranging from the frequently observed to the exceedingly rare. Plain-film radiography remains the mainstay in initial work-up. MR has some distinct advantages for further characterization and assessment of potential complications.
